# Beyond the Pill: Unveiling the Characteristics of Prenatal Micronutrient Consumption Among Hungarian Pregnant Women According to Different Levels of Adherence

**DOI:** 10.3390/nu17172732

**Published:** 2025-08-23

**Authors:** Evelin Polanek, Ferenc Rárosi, Csenge Fruzsina Béky, Regina Molnár, Gábor Németh, Hajnalka Orvos, Edit Paulik, Andrea Szabó

**Affiliations:** 1 Doctoral School of Experimental and Preventive Medicine, University of Szeged, 6720 Szeged, Hungary; 2 Department of Medical Physics and Informatics, Albert Szent-Györgyi Medical School, University of Szeged, 6720 Szeged, Hungary; rarosi.ferenc@med.u-szeged.hu; 3 Albert Szent-Györgyi Medical School, University of Szeged, 6725 Szeged, Hungary; 03bekycs@gmail.com; 4 Department of Public Health, Albert Szent-Györgyi Medical School, University of Szeged, Dóm tér 10, 6720 Szeged, Hungary; molnar.regina@med.u-szeged.hu; 5 Department of Obstetrics and Gynecology, University of Szeged, Semmelweis utca 1, 6725 Szeged, Hungary; nemeth.gabor@med.u-szeged.hu (G.N.); kokutine.orvos.hajnalka@med.u-szeged.hu (H.O.)

**Keywords:** micronutrients, folic acid, vitamin D, omega-3 fatty acids, pregnancy, adherence

## Abstract

**Background/Objectives**: There is clear evidence that maternal micronutrient deficiencies result in adverse maternal and fetal health outcomes. Therefore, corrective supplementation should be considered when dietary intake is insufficient, particularly for vitamin D (VD), omega-3 fatty acids (O3), folic acid (FA), or prenatal multiple micronutrient products (PMM). Despite its significance, intake patterns in Hungary remain largely unexplored, and evaluating adherence to recommended intake levels would be of even greater importance. This is the first Hungarian study to provide a comprehensive overview of the frequency and adherence patterns of micronutrient supplementation among pregnant women, while also analyzing their association with predictors and outcomes. **Methods**: This cross-sectional study involved 300 pregnant women who delivered in a university hospital. Data were collected using a self-reported questionnaire and clinical maternal and neonatal records. **Results**: The prevalence of FA, VD, O3, and PMM intake among the participants was 89.0%, 76.4%, 58.7%, and 67.6%, respectively. However, adherence to recommendations was notably lower: 41.1% for VD, 37.5% for O3, 36% for PMM, and 31% for FA. Higher adherence was associated with older maternal age, higher educational level, county town residence, planned pregnancy, primiparity, previous spontaneous abortion, and early initiation of antenatal care. Our findings refute concerns about the obesogenic effect of supplementation for both mothers and newborns. FA intake correlated with a lower likelihood of cesarean section, while O3 use was associated with improved uterine contractility and reduced risk of gestational diabetes mellitus. **Conclusions**: Our study underscores the need for individualized counselling on micronutrient supplementation, with particular emphasis on appropriate timing, dosage, and potential benefits.

## 1. Introduction

International guidelines on dietary supplement use during pregnancy emphasize the importance of micronutrient supplementation to improve the health outcomes of both mothers and their newborns [1]. Dietary supplement use does not substitute a balanced diet; however, it contributes to maintaining the nutrition security of pregnant women by ensuring they receive the necessary nutrients, as diet alone may not meet the increased nutritional demands during pregnancy [2,3]. Understanding dietary patterns and supplement consumption helps in designing tailored nutritional programs and interventions, particularly to address seasonal variations in dietary habits and ensure a diverse diet during gestation [4].

Micronutrient intake during pregnancy is critical for maternal health and optimal fetal development. Key prenatal micronutrients such as folic acid (FA), vitamin D (VD), omega-3 fatty acids (O3), and specific complex pharmaceutical products, the prenatal multiple micronutrient supplements (PMM), play distinct and vital roles.

FA (vitamin B9) is well-known for preventing neural tube defects. Beyond that, it is associated with a lower risk of heart defects and possibly reduces the likelihood of autism spectrum disorder in offspring [5] and has a protective role against preterm birth [6]. Both European (relevant in Hungary) and American recommendations are in line with results of systematic reviews regarding FA intake during pregnancy: 400 µg intake should be initiated at least one month before conception and continued until the end of the first trimester [7,8,9,10]. The Hungarian reproductive-age women’s dietary intake is much less than the suggested dose (only 133 µg); therefore, supplementation is strongly recommended for them [11].

VD supply during pregnancy is crucial in preventing adverse outcomes such as rickets, low birth weight, and fetal bone deformity. It also plays a role in reducing the incidence of preeclampsia and gestational diabetes (GDM) in mothers [12] and can also affect uterine contractility [13]. Deficient VD status can increase the likelihood of prolonged labor or the abnormal rupture of membranes, both of which could necessitate cesarean section [14,15]. Antenatally, a daily 600 IU (15 µg) oral VD intake is suggested generally according to international guidelines [8,16,17]; however, the World Health Organization (WHO) only recommends 200 IU (5 µg) per day and just for pregnant women with suspected VD deficiency [18]. Based on physiology, it is necessary to consume in all three trimesters. Both the Hungarian and WHO recommendations propose the same vitamin D intake levels as for the non-pregnant population. However, while the WHO emphasizes the role of dietary sources and sunlight exposure, the Hungarian national guideline recommends a higher intake, including supplementation. Average Hungarian nutritional input is extremely low, only 2.1 µg (84 IU) [11]; therefore, the Hungarian Consensus Recommendation advises 2000 IU (50 µg) for pregnant women [19].

Long-chain O3 (eicosapentaenoic acid [EPA] and docosahexaenoic acid [DHA]) are important for fetal brain development and may influence gestational length. Moreover, O3 consumption can help to control the maternal lipid profile and reduce the risk of GDM and preeclampsia [20]. O3 enhances the development of the fetal and infant brain and is important for overall health [21]. O3 is typically administered from the 20th week of gestation in a dose of 250 mg DHA + EPA plus 100 mg DHA [22,23]. Coletta et al. [24] suggest that intake throughout the whole pregnancy is advantageous. Previously, the protocols advised that the administration of O3 should be stopped around the 37th week of gestation due to vasodilation and bleeding; however, a Cochrane review refuted it [25]. According to the national data, average Hungarian adult women ingest 160 mg EPA + DHA by nutrition, leading to a need for supplementation [11]. There is no official Hungarian recommendation for O3 supplementation for pregnant women.

Several recommendations exist regarding PMM supplementation during pregnancy, although not in Hungary. PMM with 13–15 components (including 30 mg iron and 400 µg FA) are recommended during pregnancy by the WHO [26]; hence, this suggestion is mainly targeted to low- and middle-income countries. Although the duration of intake is not specified, regarding the health effects of each component, it is advised to consume throughout the entire pregnancy. It is further supported by the fact that the Hungarian adult population is in shortage of many micronutrients, as revealed by the recent National Nutritional Status Study [11]. Although PMM were previously linked to being large for gestational age due to cephalopelvic disproportion, later this was not confirmed [27].

The prevalence of dietary supplement use among pregnant women varies significantly across different regions and populations, but it is generally high. In Iran, 69% [2]; in Japan, 75% [28]; in Saudi Arabia, 71.5% [29]; in China, 94.8% [30]; in the United States, 78% [31]; in the Netherlands, 64.8% [32]; and in Canada, 90% of pregnant women reported using dietary supplements [33]. Previously, in Hungary, mainly FA supplementation was studied: at the beginning of the 2000s, 69% of the pregnant women took FA [34,35], while in 2006 it was 40% [36]. Currently there is no information about the pattern of either FA or about other maternal supplementation in Hungary. Moreover, information about obedience to guidelines, the proper doses, and the proper duration, as well as adherence, would be important aspects to know.

Previous studies showed that several sociodemographic and obstetric characteristics are associated with dietary supplement use among pregnant women. Maternal age, level of education, family income, and history of miscarriage have been identified as significant factors influencing dietary supplement consumption during pregnancy [2]. Additionally, maternal age and parity, especially among pregnant women with obesity, are also associated with dietary supplement use [37].

Currently in Hungary the number of dietary supplements on the market is more than 25,000. This extremely high number is due to a lack of approval procedure. Moreover, no special limitations are applicable for dietary supplements used among vulnerable populations, like pregnant women or children [38]. Thus, Hungarian pregnant women favor PMM products. Finding the appropriate dietary supplements among the huge variety of options requires health consciousness and knowledge regarding the required nutrients. Nonetheless, pregnant women’s knowledge regarding dietary supplement use varies and is often inadequate in certain aspects, particularly concerning the appropriate timing and doses, in addition to the importance of each individual nutrient. Sato et al. [28] found that pregnant women start using supplements containing FA only after recognizing their pregnancy. Hungarian research revealed that 93% of pregnant women who took FA started the supplementation only after the seventh week of gestation, when the neural tube development has already been completed [34]. Only about 55% of women in their first trimester of pregnancy take a supplement containing FA. This low percentage suggests limited knowledge or adherence to recommendations for early pregnancy supplementation [31].

The aim of our study was to assess the Hungarian pregnant women’s dietary supplement consumption by focusing on the most important micronutrients. Due to the lack of information in this field, we wished to obtain an up-to-date and comprehensive database. The study analyzes the intake patterns and their sociodemographic, obstetric, and lifestyle characteristics; explores the level of adherence to guidelines; and investigates the predictors of inappropriate and appropriate use. Moreover, the study analyzes the possible correlations between dietary supplement intake and pregnancy outcomes among pregnant women who delivered in Szeged, Hungary.

## 2. Materials and Methods

### 2.1. Study Design and Participants

With the collaboration of the Department of Obstetrics and Gynaecology and the Department of Public Health, University of Szeged, a questionnaire-based study, the “Quantifying Maternal Non-Obstetrical Risk Factors for Preterm Birth—Retrospective and Prospective Study”, was performed. Present results are based on the data of the retrospective case–control branch of the study. The opportunity for participation was offered to each adult (>18 years) woman who had a preterm birth in 2019, during March–December, at the university hospital of Szeged; these mothers made up the case group. After every included preterm birth, two consecutive term deliveries were included as control cases, adjusted to maternal age and date of delivery. Multiple pregnancies was an excluding criterion in both groups. Altogether, 300 women were included in the study: 100 preterm and 200 term pregnancies. In the present analysis, both cases and controls were analyzed together in a secondary outcome model focusing on dietary supplement consumption and lifestyle factors of included women.

### 2.2. Study Variables

The dataset compilation was predicated on a dual-faceted approach: a self-reported questionnaire complemented by an examination of maternal and neonatal health records. The questionnaire included a comprehensive array of data encompassing demographic and socio-economic data, lifestyle data, obstetric history, and information regarding current pregnancy and dietary supplement intake. Although the questionnaire applied in this study was not formally validated, a small-scale pilot test was conducted prior to data collection in order to detect potential sources of bias and to refine the instrument’s design. Concurrently, in order to minimize the recall bias of the questionnaire, health documentation of included women was also evaluated, which comprised a spectrum of maternal health characteristics, including the antenatal period and the immediate postpartum phase; data about delivery type and complications; and the health characteristics of the newborn.

Maternal sociodemographic status included maternal age, educational level, type of residence, and relationship status. We also examined whether the present pregnancy was the woman’s first pregnancy; whether she planned the pregnancy; and whether she had any preceding pregnancy complications, including spontaneous abortion (miscarriage) or preterm delivery. The week of the first visit at pregnancy care was also evaluated (categorized as before or after the 12th week of gestation); nonetheless, we asked the women whether they obtained any information about healthy lifestyles during pregnancy, and if yes, what was the source of information (medical professional or not). A question about more conscious dietary habits during pregnancy was also inserted into the questionnaire.

Body weight gain during pregnancy was calculated by using the reference categories of the Institute of Medicine, USA [39]. If the weight gain compared to the pre-pregnancy body mass index was lower than recommended, then it was categorized as “less than optimal”; if within the recommended range, then it was categorized as “normal”; and if more than the recommendation, then it was categorized as “more than optimal”.

Dietary supplement consumption included questions regarding PMM intake and “separate” FA-, O3-, and VD-containing dietary supplement intake, either in the form of PMM or products with a single component. The start and end weeks of consumption of these supplements were asked as well. Knowledge regarding the components of the used PMM was evaluated in connection with FA, omega-3, and VD content. Micronutrient intake was determined by analyzing the ingredients of the reported PMM and other products.

For characterizing the consumption pattern and adherence of PMM, FA, VD, and O3, three categories were created for each of them: appropriate users, inappropriate users, and non-users. Appropriate use for PMM meant that the pregnant women started to take them from at least the 9th week of gestation until delivery; for FA, women started to take them before getting pregnant until at least the end of the first trimester (12th week of gestation); for VD, from at least the 9th week of gestation until delivery; and for O3, from at least the 20th week of gestation until delivery. Though ideally VD or PMM should be provided throughout the whole pregnancy, expecting women usually initiate their use after recognizing and confirming their pregnancy. The choice of the 9th week as the initiation point was based on the practice in Hungary that the first prenatal care visit with ultrasound to notify the pregnancy and visiting the designated midwife must be done ideally until the 8th week of gestation. Assuming that advice on micronutrient intake is provided during these visits, pregnant women should begin taking VD or PMM at least from this point onward. The 20th week as the recommended starting point for appropriate adherence to O3 is supported by the EFSA Scientific Opinion [22] and by a clinical practice guideline endorsed by several international health societies [23]. Inappropriate users were those who took PMM or respective micronutrients, but differently from the “appropriate” category. Non-users were those who absolutely did not supplement PMM or single micronutrients.

Based on the previous classifications, a composite adherence score was also calculated for FA, VD, and O3 together, taken from any sources (from PMM or a single-component product). A total of 2 points were given for each appropriate user, 1 point for each inappropriate user, and 0 points for each non-user; altogether, a maximum of 6 points could be achieved. According to adherence scores, 3 categories were formed: if the total score was 5–6, they were the appropriate combined micronutrient users; if 2–4 points, they belonged to the inappropriate combined micronutrient users; and 0–1 points meant that they were micronutrient non-users.

Outcome measures of the analysis included diseases diagnosed during pregnancy (gestational high blood pressure and GDM data collected from medical reports). Maternal weight, preeclampsia, premature birth, weight of the newborn, and delivery complications such as inadequate uterine contraction, cephalopelvic disproportion, and abnormal membrane rupture were also assessed as outcome measures.

### 2.3. Statistical Analysis

Characteristics of the study population, supplementation sources, and intake appropriateness (adherence) were evaluated by descriptive statistics. The relationships between dietary supplement use and socioeconomic or basic characteristics were assessed with chi-square tests. Looking for predictors of different adherence levels was conducted by multinomial logistic regression analysis, where dependent variables were the three categories of micronutrient adherence levels. The predictors (independent variables) included were parity, previous spontaneous abortion, first appearance at prenatal care, advice about pregnancy, and attention to diet and weight gain during pregnancy. The association between the adherence to supplementation recommendations and pregnancy-, delivery-, or birth-related outcomes was carried out by binary logistic regression through the enter method. Here, dependent variables comprised GDM, gestational hypertension, preterm birth, high birth weight, inadequate uterine contraction, cephalopelvic disproportion, abnormal rupture of membranes, and cesarean section. We considered a result significant if *p* < 0.05 in the case of each statistical method. For regression analyses, the odds ratios (ORs) and their corresponding 95% confidence intervals (CIs) were also calculated for each variable. Statistical analysis was performed by using the IBM SPSS 29.0 program.

### 2.4. Ethical Approval

The study protocol was approved by the Regional and Institutional Human Medical Biological Research Ethics Committee of the University of Szeged, Hungary. Approval reference number: 4419; 256/2018-SZTE. Approval date: 10 December 2018. The participation was voluntary, and a written informed consent was obtained from each participant.

## 3. Results

### 3.1. Study Population

The main characteristics of the study population and the distribution of the investigated variables are seen in Table 1. The average age of the included women was 32 years, and 42.4% of the mothers were older than 35 years. A total of 41.5% of the mothers had a university degree, 97.6% lived in a relationship, and 45% settled in a county town at the time of filling out the questionnaire. The most frequent educational level of the fathers was secondary school (53.1%). In 80.8% of the cases, the pregnancy was reported as planned. In 41.5% of the women, the current pregnancy was the mother’s first pregnancy. The majority of the pregnant women attended their first visit at pregnancy care before the 12th week of gestation (83.7%), 73.3% received advice regarding pregnancy and healthy lifestyles from a medical professional, and 77.6% paid more attention to diet during pregnancy. Almost a quarter of the participants (23.2%) had a previous spontaneous abortion. A third of the women gained body weight during pregnancy within the normal range, while 39.3% put on more than the recommendation.

### 3.2. Dietary Supplement Consumption

#### 3.2.1. Sources of Dietary Supplements

Among the total study population, the most commonly reported method of micronutrient intake was PMM use, with 69.0% of participants indicating regular consumption during pregnancy. Products generally consisted of 12–15 components, but not all of them contained FA, VD, or O3. Regarding individual micronutrients, 77.6% of women took VD, 88.7% used FA, and 60.1% reported omega-3 supplementation. While a substantial number of participants relied solely on multivitamin complexes, combined usage patterns were also present: 23.7% took FA from PMM and from other sources; 13.5% of respondents used both PMM and additional VD products, and 10.8% used both PMM and separate O3 supplements. Altogether, 88.7% of the pregnant women supplemented FA, 77.6% VD, and 60.1% O3 during pregnancy (Table 2).

#### 3.2.2. Micronutrient Intake Appropriateness, Adherence to Guidelines

**Figure 1:**
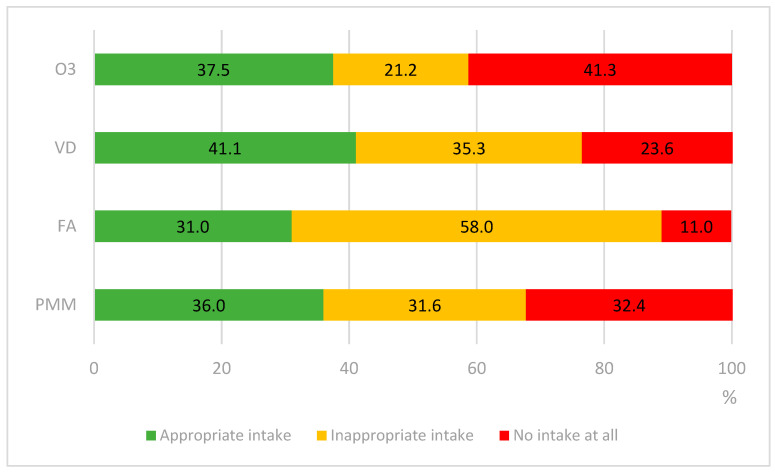
Micronutrient intake appropriateness and adherence to guidelines. Abbreviations: O3: omega-3 fatty acids; VD: vitamin D; FA: folic acid; PMM prenatal multiple micronutrient supplements.

Adherence to proper intake varies widely regarding each micronutrient. The study population showed the highest adherence to VD (41.1%), followed by O3 (37.5%), PMM (36%), and FA (31%). However, 58% of FA consumers did the supplementation but in an inappropriate way. Incorrect adherence to VD, PMM, and O3 was detected in 35.3%, 31.6%, and 21.2% of the study population, respectively. Nonetheless, a notable proportion reported no supplementation at all—32.4% did not take PMM, 11.0% did not take FA, 23.6% did not use VD, and 41.3% abstained from O3 (Figure 1).

**Figure 2:**
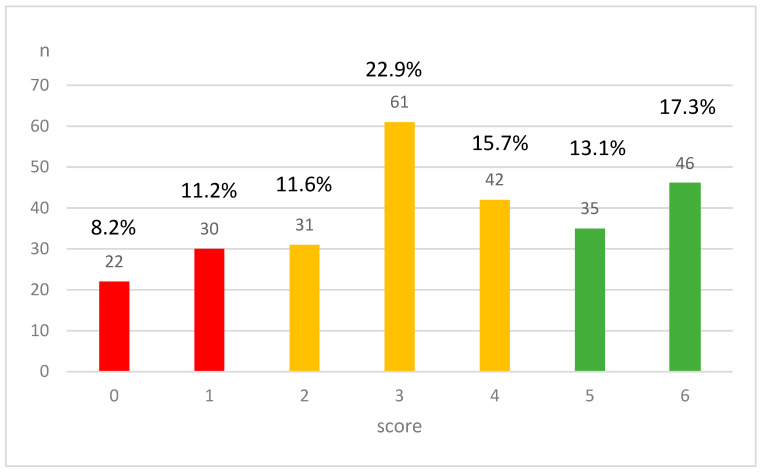
Distribution of the composite adherence score values for combined micronutrient intake (FA + VD + O3). Red: almost non-users, yellow: inappropriate users, green: appropriate users. *n* = number of cases.

Analyzing the composite adherence score (Figure 2), it can be seen that 8.2% of the study population did not take FA, VD, or O3 (score 0), and another 11.2% of the women took just one of the three micronutrients, but incorrectly. Altogether, 50.2% of the pregnant women achieved scores of 2–4, meaning that they took at least one micronutrient correctly, two of them incorrectly, or a maximum of two of them were administered appropriately. The highest scores of 5–6 were achieved by 30.4% of the included women, indicating that, at minimum, they supplied two of the micronutrients correctly and one incorrectly, or all of them were taken appropriately.

### 3.3. Adherence to Micronutrients

#### 3.3.1. Sociodemographic Characteristics of the Different Adherence Groups

Descriptive analyses revealed significant associations between micronutrient intake and several sociodemographic characteristics (Table 3, Table 4, Table 5, Table 6 and Table 7). Maternal age was significantly associated with supplement adherence, particularly in relation to FA intake (*p* = 0.004) and combined supplement correctness (*p* = 0.032). Women under the age of 29 were less likely to take supplements correctly compared to older age groups. Educational attainment emerged as another strong predictor. University-educated women demonstrated significantly higher rates of correct FA (*p* < 0.001), O3 (*p* = 0.006), and combined supplement intake (*p* = 0.005). Place of residence was significantly associated with FA use (*p* = 0.007), with higher adherence among women living in county towns compared to smaller settlements. Marital status or the fathers’ educational level did not show association with any consumption pattern. There was no difference between PMM or VD intake categories and sociodemographic characteristics. Planned pregnancy was strongly associated with correct intake of all examined micronutrients. Participants who reported planning their pregnancies were significantly more likely to consume PMM (*p* = 0.004), FA (*p* < 0.001), or VD (*p* = 0.006). Moreover, these women exhibited higher adherence across all three supplements combined (*p* = 0.004).

#### 3.3.2. Predictors of Adherence to Micronutrients

According to the multinomial logistic regression analyses, only parity seemed to marginally influence the appropriate use of PMM compared to non-users (OR = 2.10, *p* = 0.053) (Table 8).

Correct VD intake was significantly more frequent (OR = 3.37, *p* = 0.004) among those pregnant women who were expecting their first child, compared to multiparous ones. If the first appearance at prenatal care happened before the 12th week of pregnancy, it increased the likelihood of more conscious VD supplementation either in an inappropriate (OR = 2.53, *p* = 0.039) or appropriate way (OR = 2.75, *p* = 0.023), compared to those not taking VD (Table 9).

In the case of primigravida women or those with a history of previous spontaneous abortion, the odds of appropriate O3 intake were 2.21 and 2.66 times higher, respectively (*p* = 0.005 and 0.043, respectively), compared to non-users (Table 10).

The adherence to correct FA intake during pregnancy was 3.16- and 4.7-times higher in the case of first parity (*p* = 0.04) or when the first prenatal care occurred before the 12th week of gestation (*p* = 0.009). In the latter case, starting the prenatal care early also led to the supplementation of FA but in an inappropriate way, although almost below the level of significance (Table 11).

Looking for the predictors of proper combined micronutrient intake, there were 5.23 times higher odds associated with being pregnant for the first time (*p* = 0.001) compared to non-users and multiparity. Starting prenatal care early was definitely a positive predictor of taking micronutrients, both for incorrect (OR = 3.57, *p* = 0.006) or correct (OR = 3.75, *p* = 0.012) intake (Table 12). Correct (OR = 2.93, *p* = 0.044) or nearly correct (OR = 3.18, *p* = 0.020), combined users were more likely to gain normal weight during pregnancy. Such an association was not detected in the case of supraoptimal weight gain.

#### 3.3.3. Association of Micronutrient Supplementation with Different Adherence Levels on Pregnancy, Delivery, and Birth

Regarding pregnancy-related outcomes (Table 13), gestational hypertension was not linked to any supplementation. Nevertheless, there were significantly lower odds for GDM in the case of appropriate O3 intake (OR = 0.06, *p* = 0.047) and unlikely in the case of inappropriate supplementation, compared to O3 non-users. O3 correlated with delivery outcome as well by increasing 4.24-fold the chance of inadequate uterine contraction (*p* = 0.005); however, correct use of O3 was not proven significant (Table 14). Both inappropriate and appropriate use of FA were associated with a decreased occurrence of cesarean section at 69% and 75%, respectively (*p* = 0.038 and *p* = 0.036, respectively, Table 14). Other delivery- and birth-related events, such as preterm birth, high birth weight, cephalopelvic disproportion, and abnormal membrane rupture, were not associated with any micronutrient intake.

## 4. Discussion

In our study the prevalence of micronutrient supplementation, adherence to guidelines, the predictors of good adherence, and the association of micronutrient supplementation with different adherence levels on pregnancy, delivery, and birth were investigated. The prevalence of FA, VD, and O3 intake among the participating pregnant women was 88.7%, 77.6%, and 60.1%, respectively. A total of 69% of participants consumed PMM, 8–21% supplemented only from other sources (mainly single-component products, not PMM), and 10–23% supplemented both from PMM and other sources. Roughly 30—40% of pregnant women supplemented the micronutrients appropriately: 41.1% for VD, 37.5% for O3, 36% for PMM, and 31% for FA. Another 20–50% of pregnant women took the micronutrients in an inappropriate way, especially FA at 58%. Almost half of the expectant women did not intake O3, though it was proven beneficial to relieve inadequate uterine contraction or prevent GDM by properly administering it. A total of 30% of the included population supplemented all three micronutrients correctly. Among population characteristics, older maternal age, higher educational level, and living in the county town appeared to result in high adherence, especially in the case of FA. Planned pregnancy was also more frequent in high adherence groups in the case of almost all the investigated micronutrient groups. Predictors of good adherence consisted of primiparity and an earlier visit to antenatal care, but mainly if the micronutrient was supplemented appropriately. Combined supplement users were more likely to gain normal weight during pregnancy; nonetheless, this did not lead to high birth weight. FA intake correlated with lower odds of requiring a cesarean section.

Adequate micronutrient intake during pregnancy is a well-documented prerequisite for healthy maternal and fetal outcomes. Despite decades of scientific consensus and public health guidelines, multiple international studies indicate that a considerable proportion of pregnant women still do not meet the recommended intake levels for key nutrients such as FA, VD, O3, iodine, and iron [40]. While many national guidelines recommend the use of PMM that contain a broad array of micronutrients, intake patterns among pregnant women show remarkable heterogeneity across countries and social groups.

International cohort data suggest that the majority of pregnant women in high-income countries rely on PMM, although not uniformly. In the United States, Jun et al. [41] found that 77% of pregnant women used dietary supplements, predominantly multivitamins, with 53% taking FA separately. A similar trend was reported in Canada, where over 80% of pregnant women used supplements, and approximately 60% took single FA products [42]. In contrast, a Swedish cohort study by Bärebring et al. [43] noted that 78% of women used some form of supplementation during pregnancy, but only 43% specifically took VD, and 25% used O3. This pattern is similar to ours, where intake of O3 remains underused, despite growing evidence supporting its role in fetal brain development [44]. In our study, PMM use was common, but intake of individual micronutrients varied substantially. Importantly, only a minority relied on separate supplements: for example, only 8.8% used VD exclusively, while 13.5% took both PMM and an additional VD-containing product. Thus, although most women reported some form of supplementation, the pattern suggests that multivitamin use alone does not guarantee optimal intake of all required nutrients, especially when dose adequacy is uncertain or the content is not well understood by the users.

These findings have important implications for maternal health policy and clinical practice. To improve adherence to micronutrient supplementation guidelines, national antenatal care protocols should prioritize early prenatal visits as a critical period for intervention. Integrating targeted counselling into the first antenatal visit—particularly for younger, less educated, or multiparous women—may significantly enhance understanding and compliance with recommended intake levels. Healthcare providers should be equipped with concise, evidence-based guidance on the benefits of FA, VD, and O3, with emphasis on the timing, dosage, and formulation (e.g., PMM vs. single-component supplements). Furthermore, routine antenatal care could include personalized supplementation plans based on maternal characteristics such as parity, educational background, and residence. Public health campaigns and digital health tools (e.g., mobile reminders, educational apps) may further support behavioral change and address knowledge gaps. These structural enhancements could help close the gap between recommendations and actual practices, thereby promoting healthier pregnancy outcomes on a population level.

FA supplementation in the past two decades shows some improvements. In our cohort, 89.0% of the pregnant women supplemented FA, while previously it was only 40% or 69% in Hungary [34,36]. Although comparable adherence data are not available, the preceding Hungarian studies found that only 7–30% of the women started to take FA before conception [34,36]. Assuming that they are the so-called appropriate users in our study, this has not changed a lot over time, as in our cohort also just 31% of women initiated supplementation before conception. Malek et al. [45] had very similar findings: 27% of pregnant women took FA correctly. The results were also comparable in a German study [46], where high prevalence of FA supplementation was reported with much lower adherence (36.2%) to recommendations.

Interestingly, a 2013 multi-national study by Aronsson et al. [47] highlighted that multivitamin adherence is often high in high-income countries but varies significantly by socio-demographic subgroups. Our findings also support this variability: although general use is high, descriptive statistics emerged that age and education are robust contributors of correct supplementation; younger women and those with lower education were significantly less likely to follow correct supplementation practices. Women under 29 were less prone to correctly use FA compared to those over 35. This is consistent with findings from the United Kingdom [48], where young maternal age was linked to lower supplement adherence. The results are in line with earlier studies as well, linking lower maternal age with reduced health literacy, inconsistent antenatal care attendance, and diminished awareness of dietary guidelines [49]. Education had a similarly strong effect in our study; university-educated women were significantly more susceptible to correctly using dietary supplements during their pregnancy. This aligns with the results of Haugen et al. [50], who found that supplement use in Norway was positively associated with maternal education and income. In our study, the education of the father did not seem to be influential on the mother’s supplementation practices, indicating that pregnant women must take the responsibility on their own. These findings highlight the importance of targeted education campaigns for younger and less-educated female populations. Similarly, de Paula et al. [51] emphasized that higher education correlates strongly with micronutrient awareness and intake, as higher educational attainment is correlated with increased nutritional knowledge and supplement adherence during pregnancy.

Planned pregnancy emerged as one of the most influential contributors of correct supplementation behavior. Women with planned pregnancies were significantly more likely to take every studied supplement correctly. These results mirror those of Bodnar et al. [52], who demonstrated that women who actively plan their pregnancies were associated with positive health behaviors, including appropriate nutrient intake, and are more likely to engage in higher PMM use and earlier prenatal care initiation in the U.S.

Among the examined predictors of appropriate micronutrient intake, some were significantly associated with either regular but inappropriate intake or with correctly following the recommendations and thus achieving appropriate intake. These findings underscore the importance of not only promoting supplement use during pregnancy counselling but also ensuring that women have adequate knowledge and guidance to follow dosage and timing recommendations correctly.

The reasons for non-adherence identified in our study can partly be explained by sociodemographic and pregnancy-related factors. Additional determinants, such as cultural influences or health policy implications, should be considered in future Hungarian research on maternal adherence to supplementation. International studies have highlighted several behavioral and socio-political barriers beyond sociodemographic parameters, including lack of time, forgetfulness, high cost, fear of side effects, limited awareness of importance, difficulty in tablet intake, poor access to prenatal care services, and inadequate counselling [53,54,55]. Furthermore, Doru et al. [46] reported that adherence was more likely among women following a vegetarian/vegan diet, intending to breastfeed, or regularly consuming medications.

According to our results, parity significantly increased the occurrence of appropriate supplementation of all studied micronutrients, alone or in combination, but did not increase the chance for improper supplementation, but at least they were taking the micronutrients. Consistent with our result, Moser et al. [56] found that multiparity is associated with inappropriate FA intake during pregnancy. These results may suggest that nulliparous mothers are more conscious and receive greater attention during pregnancy care, possibly due to their status as new and inexperienced mothers. When analyzing serum VD status, Yang et al. [57] found that parity of more than two children is significantly associated with lower VD levels, which underlines the need for more attention in the case of multiparous pregnancies. Nonetheless, Polanek et al. [58] found that dietary VD intake is insufficient and it is not associated with maternal serum VD levels, highlighting the importance of dietary supplement use.

In our study the history of pregnancy loss also emerged as a positive predictor of appropriate O3 supplementation. Pregnant women with prior miscarriage may be encouraged to intake O3 since recent scientific evidence reinforces that O3 plays a significant role in improving reproductive outcomes by several physiological processes, including improved lipid levels, enhanced insulin sensitivity, inhibited pro-inflammatory pathways, ameliorated oxidative stress, and thyroid dysfunction, thus leading to boosted capacity for implantation and fetal development. Moreover, researchers also raise the possibility of applying O3 in the treatment of recurrent miscarriages [59].

Early presentation at prenatal care (before the 12th week of gestation) was also associated with several micronutrient intakes: it increased the likelihood of VD intake (both appropriately and inappropriately), FA intake (appropriately), and combined intake (both appropriately and inappropriately). Emphasizing the importance of pregnancy counselling, Polanek et al. [60] found that early presentation at pregnancy care indicates a significantly higher maternal health promoting behavior index and, therefore, a higher overall health consciousness, and Le et al. [61] found that limited health literacy was associated with the lowest supplement use among the study population. Previous studies have found that recommendations from healthcare professionals during pregnancy care are strongly associated with supplement use [62]; however, the appropriateness of consumption has been less frequently evaluated. In contrast, according to our results, advice from a medical professional regarding a healthy lifestyle or paying more attention to diet during pregnancy did not seem to be a predictor of correctly using the studied micronutrients, despite the fact that three-fourths of the participants responded that they obtained information from a health care professional. This raises the possibility of ineffective communication techniques during counselling. The systematic review of Gomes et al. [53] identified several successful interventions designed to increase adherence to prenatal micronutrient supplementation (education-based strategies, consumption monitoring, SMS reminders, or free provision of supplements) that can be adopted in Hungary as well. A further deficiency of the Hungarian antenatal care is identified by our results, as in smaller settlements than the county town (Szeged), FA supplementation was less correct. This reveals an imbalanced implementation of national recommendations on maternal FA supplementation. The reason for this is unknown; more research is needed in this specific area. Nonetheless, prenatal care in Hungary includes gynecologists and midwives together with general practitioners. Their tasks are regulated by laws; a decree about pregnancy care includes a paragraph that the caregiver should pay special attention to the intake of FA and VD during pregnancy and informs the pregnant woman about the harmful effects of FA deficiency on the fetus [63]. No other professional guideline mentions supplementation, nor is any other micronutrient recommended officially in Hungary. This is an area to update in the Hungarian antenatal care.

According to our study, healthier weight gain during pregnancy was associated with the combined supplementation of FA, VD, and O3. This finding contradicts common preconceptions among pregnant women suggesting that dietary supplementation during pregnancy leads to excessive weight gain and a bigger fetus. This fear is mirrored in our results as well, as pregnant women with higher weight gain than the recommended did not appear to consume any micronutrients with higher odds. Tabrizi et al. [64] found that dietary supplementation reduces the risk of inadequate weight gain during pregnancy, while Alwan et al. [65] reported no association between supplement use and birth size. Our results also refuted the theory by not finding a connection between any supplement use and a high birth weight.

The association between neonatal and obstetric outcomes and different adherence levels of micronutrient intake was also evaluated. The effect of micronutrients on pregnancy was manifested on one hand in the protective effect of O3 intake against GDM, though only by correct usage. Evidence from recent randomized controlled trials and reviews also underscores that O3 can improve insulin sensitivity and glycemic control, therefore being able to reduce fasting blood glucose and, moreover, reducing inflammatory responses in women with GDM [66,67].

On the other hand, during delivery, O3 intake appeared as a risk factor for inadequate uterine contraction, but only if administered inappropriately. Physiologically, O3-derived lipids may influence vascular smooth muscle activity and trophoblast functions within the placenta, potentially mediating uterine contraction dynamics [68]. They can also modulate the production of prostaglandins, potentially leading to a delay in uterine contractions [69]. Finally, they can prevent premature contractions and can prolong the pregnancy as a favorable effect, though this can be unwanted during labor [59].

A notable finding of our study was the significant protective association between FA supplementation and a reduced likelihood of cesarean section. Although directly investigating the relationship between FA and cesarean sections is very rare in the scientific literature, Meena et al. [70] found no significant association between maternal serum FA level and mode of delivery. Nevertheless, several explanations exist for understanding the indirect role of FA: promotion of cell growth and thus better fetal health outcomes and reduced risks of complications such as placental abruption, low birth weight, preeclampsia, and preterm birth, therefore extending gestational age and potentially reducing emergency cesarean deliveries [71,72,73]. Nevertheless, Cheng et al. [74] revealed that adequate FA supplementation for more than 3 months before and during pregnancy might decrease the effect of GDM on macrosomia, thereby not increasing the risk of cesarean delivery.

This study also had some limitations. Due to the cross-sectional nature of the study, causation cannot be deduced, and biases may impact the results. The participation in the study was offered for all eligible women, but the final decision about the enrollment was given by the participants (selection bias). Data regarding pregnancy were collected after delivery, and no formal validation of the questionnaire was conducted. Postpartum data acquisition introduces recall bias, as the self-administered modality of the survey could skew responses due to selective memory or the inclination to portray oneself favorably (social desirability bias). The investigation was conducted within a single institution; nevertheless, it has regional responsibility, potentially enhancing the applicability of the findings. Dietary intake was not assessed for FA and O3; thus, total micronutrient exposure could not be determined. This limits the interpretation of supplementation effects in isolation. Notwithstanding these impediments, the analysis provides a comprehensive examination of the dietary supplement consumption habits of pregnant women, recently the only one available in Hungary.

## 5. Conclusions

To the best of our knowledge, this is the first study in Hungary that not only measures the prevalence of supplement consumption but also examines the adherence of pregnant women to micronutrient supplementation guidelines. Our research shows that the majority of pregnant women in Hungary do not meet micronutrient recommendations: despite a relatively high prevalence of supplement use (60–89%), adherence to guidelines (i.e., correct use) is low (30–40%), while 21–58% of women take micronutrients incorrectly, even though most pregnancies are reported as planned. The data regarding FA supplementation practices were the most concerning of all. These findings underscore the urgent need to disseminate dosage- and timing-specific dietary supplement recommendations through effective nutritional counselling during pregnancy care and within family planning services. The adoption of appropriate strategies, clear communication, and reliable information sources at the national level would play a crucial role in increasing adherence to recommended micronutrient intake during pregnancy.

Overall, our findings highlight the complexity of micronutrient intake behaviors among pregnant women: sociodemographic factors, parity, previous pregnancy history, timing of the first antenatal care visit, and weight gain during pregnancy all influence the level of adherence. Finally, appropriate intake of FA and O3 is clearly associated with improved pregnancy and delivery outcomes, whereas these benefits are not observed in cases of incorrect micronutrient consumption—further emphasizing the importance of adherence to proper recommendations. These insights offer concrete directions for both public health interventions and policy development. Public health campaigns should aim to raise awareness about timing-specific and evidence-based supplementation practices, with tailored messaging for at-risk populations. Moreover, the findings could support updates to Hungarian antenatal care guidelines, encouraging the integration of structured micronutrient counselling at early prenatal visits and during preconception planning. Establishing standardized counselling protocols and monitoring tools within national maternal care systems may significantly improve adherence and ultimately pregnancy outcomes.

## Figures and Tables

**Table 1:** Characteristics of the study population.

Characteristics	*n*	%
Demographic characteristics		
Age group (years)		
≤29	73	24.3
30–34	100	33.3
≥35	127	42.4
Education of mother		
primary	25	8.5
secondary	97	33.0
higher-level vocational training	50	17.0
university	122	41.5
Education of father		
primary	16	5.5
secondary	155	53.1
higher-level vocational training	31	10.6
university	90	30.8
Marital status		
single	7	2.4
in a relationship	290	97.6
Residence		
capital city, county town	134	45.0
city	89	29.9
village	75	25.1
Pregnancy-related characteristics		
Planned pregnancy		
yes	235	80.8
no	56	19.2
Parity		
first	124	41.6
second or more	174	58.4
Timing of first visit at pregnancy care		
≤12 weeks	241	83.7
>12 weeks	47	16.3
Advice regarding lifestyle during pregnancy		
from a medical professional	211	73.3
from a non-medical source	77	26.7
Previous spontaneous abortion		
yes	69	23.2
no	228	76.8
More attention on diet during pregnancy		
yes	228	77.6
no	66	22.4
Body weight gain during pregnancy		
More than optimal	116	39.3
Normal	90	30.5
Less than optimal	89	30.2
GDM		
yes	45	15.2
no	251	84.8
Gestational hypertension		
yes	32	10.8
no	264	89.2
Delivery- and birth-related characteristics		
Preterm birth		
yes	100	33.3
no	200	66.7
Birth weight		
<2500 g	64	21.6
2500–4000	207	70.0
>4000	25	8.4
Inadequate uterine contraction		
yes	93	31.6
no	201	68.4
Cephalopelvic disproportion		
yes	22	7.5
no	272	92.5
Abnormal rupture of membranes		
yes	105	64.5
no	191	35.5
Cesarean section		
yes	160	53.7
no	138	46.3

Note: Frequencies and percentages may not equal the total or may not add to 100% due to missing data.

**Table 2:** Sources of dietary supplements.

	Intake			
	Only from PMM	Only from Other Sources	Both from PMM and Other Sources	Altogether
	*n* (%)	*n* (%)	*n* (%)	*n* (%)
PMM	205 (69.0)	-	-	205 (69.0)
FA	131 (43.7)	64 (21.3)	71 (23.7)	266 (88.7)
VD	163 (55.3)	26 (8.8)	40 (13.5)	229 (77.6)
O3	106 (35.8)	40 (13.5)	32 (10.8)	178 (60.1)

O3: omega-3 fatty acids; VD: vitamin D; FA: folic acid; PMM: prenatal multiple micronutrient supplements.

**Table 3:** Characteristics of women taking PMM according to different adherence levels of supplementation.

Characteristics	PMM (Correctly Used)	PMM (Incorrectly Used)	PMM (Non-Users)	*p* Value
	*n* (%)	*n* (%)	*n* (%)	
Age group				
≤29	16 (25.0)	24 (37.5)	24 (37.5)	0.142
30–34	35 (37.6)	33 (35.5)	25 (26.9)	
≥35	49 (40.5)	31 (25.6)	41 (33.9)	
Education of the mother				
primary	4 (19.0)	7 (33.3)	10 (47.6)	0.274
secondary	32 (35.2)	27 (29.7)	32 (35.2)	
higher-level vocational training	13 (28.3)	16 (34.8)	17 (37.0)	
university	49 (42.2)	37 (31.9)	30 (25.9)	
Education of the father				
primary	6 (42.9)	5 (35.7)	3 (21.4)	0.209
secondary	49 (34.3)	41 (28.7)	53 (37.1)	
higher-level vocational training	6 (20.0)	11 (36.7)	13 (43.3)	
university	35 (42.2)	28 (33.7)	20 (24.1)	
Marital status				
single	3 (33.3)	3 (33.3)	3 (33.3)	0.982
in a relationship	97 (36.5)	84 (31.6)	85 (32.0)	
Residence				
county town	42 (34.1)	44 (35.8)	37 (30.1)	0.547
city	31 (36.0)	27 (31.4)	28 (32.6)	
village	27 (39.7)	16 (23.5)	25 (36.8)	
Planned pregnancy				
yes	89 (40.6)	62 (28.3)	68 (31.1)	0.004
no	9 (17.0)	24 (45.3)	20 (37.7)	

PMM: prenatal multiple micronutrient supplements.

**Table 4:** Characteristics of women taking FA according to different adherence levels of supplementation.

Characteristics	FA (Correctly Used)	FA (Incorrectly Used)	FA (Non-Users)	*p* Value
	*n* (%)	*n* (%)	*n* (%)	
Age group				
≤29	9 (14.3)	42 (66.7)	12 (19.0)	0.004
30–34	36 (38.3)	53 (56.4)	5 (5.3)	
≥35	40 (34.2)	64 (54.7)	13 (11.1)	
Education of the mother				
primary	1 (4.8)	12 (57.1)	8 (38.1)	<0.001
secondary	18 (20.5)	58 (65.9)	12 (13.6)	
higher-level vocational training	8 (18.2)	31 (70.5)	5 (11.4)	
university	58 (49.6)	55 (47.0)	4 (3.4)	
Education of the father				
primary	0 (0)	11 (84.6)	2 (15.4)	0.078
secondary	43 (30.1)	81 (56.6)	19 (13.3)	
higher-level vocational training	9 (31.0)	16 (55.2)	4 (13.8)	
university	32 (39.0)	46 (56.1)	4 (4.9)	
Marital status				
single	1 (11.1)	5 (55.6)	3 (33.3)	0.067
in a relationship	84 (32.1)	151 (57.6)	27 (10.3)	
Residence				
county town	47 (38.8)	68 (56.2)	6 (5.0)	0.007
city	16 (19.3)	53 (63.9)	14 (16.9)	
village	22 (31.9)	37 (53.6)	10 (14.5)	
Planned pregnancy				
yes	83 (38.2)	118 (54.4)	16 (7.4)	<0.001
no	2 (3.9)	36 (70.6)	13 (25.5)	

FA: folic acid.

**Table 5:** Characteristics of women taking O3 according to different adherence levels of supplementation.

Characteristics	O3 (Correctly Used)	O3 (Incorrectly Used)	O3 (Non-Users)	*p* Value
	*n* (%)	*n* (%)	*n* (%)	
Age group				
≤29	20 (29.9)	16 (23.9)	31 (46.3)	0.240
30–34	35 (36.5)	25 (26.0)	36 (37.5)	
≥35	53 (42.4)	20 (16.0)	52 (41.6)	
Education of the mother				
primary	4 (18.2)	3 (13.6)	15 (68.2)	0.006
secondary	29 (30.9)	22 (23.4)	43 (45.7)	
higher-level vocational training	14 (29.8)	12 (25.5)	21 (44.7)	
university	60 (50.0)	23 (19.2)	37 (30.8)	
Education of the father				
primary	6 (40.0)	2 (13.3)	7 (46.7)	0.243
secondary	51 (34.7)	29 (19.7)	67 (45.6)	
higher-level vocational training	9 (29.0)	9 (29.0)	13 (41.9)	
university	42 (48.3)	18 (20.7)	27 (31.0)	
Marital status				
single	3 (30.0)	3 (30.0)	4 (40.0)	0.757
In a relationship	104 (37.8)	57 (20.7)	114 (41.5)	
Residence				
county town	56 (43.1)	31 (23.8)	43 (33.1)	0.115
city	29 (34.1)	17 (20.0)	39 (45.9)	
village	23 (31.9)	12 (16.7)	37 (51.4)	
Planned pregnancy				
yes	92 (40.7)	46 (20.4)	88 (38.9)	0.076
no	13 (24.1)	14 (25.9)	27 (50.0)	

O3: omega-3 fatty acids.

**Table 6:** Characteristics of women taking VD according to different adherence levels of supplementation.

Characteristics	VD (Correctly Used)	VD (Incorrectly Used)	VD (Non-Users)	*p* Value
	*n* (%)	*n* (%)	*n* (%)	
Age group				
≤29	21 (32.3)	25 (38.5)	19 (29.2)	0.385
30–34	43 (46.2)	33 (35.5)	17 (18.3)	
≥35	51 (41.8)	41 (33.6)	30 (24.6)	
Education of the mother				
primary	5 (22.7)	8 (36.4)	9 (40.9)	0.082
secondary	35 (38.9)	31 (34.4)	24 (26.7)	
higher-level vocational training	15 (32.6)	18 (39.1)	13 (28.3)	
university	58 (49.2)	41 (34.7)	19 (16.1)	
Education of the father				
primary	5 (35.7)	6 (42.9)	3 (21.4)	0.616
secondary	60 (41.7)	46 (31.9)	38 (26.4)	
higher-level vocational training	9 (30.0)	12 (40.0)	9 (30.0)	
university	38 (45.2)	31 (36.9)	15 (17.9)	
Marital status				
single	3 (33.3)	3 (33.3)	3 (33.3)	0.747
In a relationship	112 (41.8)	95 (35.4)	61 (22.8)	
Residence				
county town	53 (42.7)	48 (38.7)	23 (18.5)	0.093
city	31 (36.0)	34 (39.5)	21 (24.4)	
village	31 (44.9)	16 (23.2)	22 (31.9)	
Planned pregnancy				
yes	102 (46.2)	73 (33.0)	46 (20.8)	0.006
no	12 (22.6)	23 (43.4)	18 (34.0)	

VD: vitamin D.

**Table 7:** Characteristics of women taking combined micronutrients according to different adherence levels of supplementation.

Characteristics	Combined Intake (FA, VD, O3) (Correctly Used)	Combined Intake (FA, VD, O3) (Incorrectly Used)	Combined Intake (FA, VD, O3) (Non-Users)	*p* Value
	*n* (%)	*n* (%)	*n* (%)	
Age group				
≤29	13 (17.8)	23 (31.5)	17 (23.3)	0.032
30–34	29 (29.0)	30 (30.0)	13 (13.0)	
≥35	40 (31.5)	24 (18.9)	27 (21.3)	
Education of the mother				
primary	2 (8.0)	7 (28.0)	9 (36.0)	0.005
secondary	21 (21.6)	24 (24.7)	20 (20.6)	
higher-level vocational training	10 (20.0)	16 (32.0)	12 (24.0)	
university	48 (39.3)	16 (32.0)	15 (12.3)	
Education of the father				
primary	3 (18.8)	5 (31.3)	3 (18.8)	0.641
secondary	42 (27.1)	33 (21.3)	34 (21.9)	
higher-level vocational training	6 (19.4)	11 (35.5)	7 (22.6)	
university	31 (34.4)	25 (27.8)	12 (13.3)	
Marital status				
single	2 (20.0)	3 (30.0)	3 (30.0)	0.793
in a relationship	80 (27.9)	73 (25.4)	53 (18.5)	
Residence				
county town	41 (30.6)	40 (29.9)	19 (14.2)	0.184
city	21 (23.6)	22 (24.7)	19 (21.3)	
village	20 (26.7)	14 (18.7)	19 (25.3)	
Planned pregnancy				
yes	74 (31.5)	54 (23.0)	39 (16.6)	0.004
no	8 (14.3)	21 (37.5)	17 (30.4)	

O3: omega-3 fatty acids; VD: vitamin D; FA: folic acid.

**Table 8:** Predictors of appropriately/inappropriately using PMM by multinomial logistic regression.

Predictors	*n*	Inappropriate PMM Users (vs. Non-Users)		Appropriate PMM Users (vs. Non-Users)	
		aOR (95% CI)	*p* Value	aOR (95% CI)	*p* Value
Parity					
first	106	1.03 (0.50–2.13)	0.938	2.10 (0.99–4.45)	0.053
second or more	143	1		1	
Previous spontaneous abortion					
yes	59	0.51 (0.22–1.19)	0.118	1.22 (0.54–2.76)	0.634
no	190	1		1	
First appearance at prenatal care					
≤12 weeks	208	1.28 (0.56–2.90)	0.560	2.12 (0.89–5.02)	0.088
>12 weeks	41	1		1	
Advice from a medical professional					
yes	183	1.55 (0.76–3.17)	0.226	1.52 (0.75–3.09)	0.242
no	66	1		1	
More attention on diet during pregnancy					
yes	195	1.02 (0.48–2.18)	0.956	1.27 (0.59–2.74)	0.540
no	54	1		1	
Body weight gain during pregnancy					
more than optimal	96	1.34 (0.62–2.90)	0.452	1.10 (0.52–2.33)	0.809
normal	75	1.40 (0.62–3.18)	0.423	1.29 (0.58–2.85)	0.532
less than optimal	78	1		1	

aOR: adjusted odds ratio; CI: confidence interval; PMM: prenatal multiple micronutrient supplements.

**Table 9:** Predictors of appropriately/inappropriately using VD by multinomial logistic regression.

Predictors	*n*	Inappropriate VD Users (vs. Non-Users)		Appropriate VD Users (vs. Non-Users)	
		aOR (95% CI)	*p* Value	aOR (95% CI)	*p* Value
Parity					
first	107	1.57 (0.71–3.48)	0.269	3.37 (1.49–7.63)	0.004
second or more	145	1		1	
Previous spontaneous abortion					
yes	61	0.82 (0.34–1.96)	0.655	1.71 (0.72–4.09)	0.226
no	191	1		1	
First appearance at prenatal care					
≤12 weeks	211	2.53 (1.05–6.11)	0.039	2.75 (1.15–6.56)	0.023
>12 weeks	41	1		1	
Advice from a medical professional					
yes	185	1.27 (0.60–2.68)	0.530	1.60 (0.75–3.41)	0.226
no	67	1		1	
More attention on diet during pregnancy					
yes	197	1.84 (0.83–4.06)	0.134	1.70 (0.77–3.74)	0.189
no	55	1		1	
Body weight gain during pregnancy					
more than optimal	99	1.54 (0.69–3.44)	0.298	1.03 (0.46–2.29)	0.944
normal	75	2.13 (0.86–5.29)	0.104	2.01 (0.83–4.87)	0.120
less than optimal	78	1		1	

aOR: adjusted odds ratio; CI: confidence interval; VD: vitamin D.

**Table 10:** Predictors of appropriately/inappropriately using O3 by multinomial logistic regression.

Predictors	*n*	Inappropriate O3 Users (vs. Non-Users)		Appropriate O3 Users (vs. Non-Users)	
		aOR (95% CI)	*p* Value	aOR (95% CI)	*p* Value
Parity					
first	108	0.98 (0.47–2.08)	0.966	2.66 (1.35–5.28)	0.005
second or more	150	1		1	
Previous spontaneous abortion					
yes	61	0.90 (0.37–2.18)	0.818	2.21 (1.03–4.76)	0.043
no	197	1		1	
First appearance at prenatal care					
≤12 weeks	215	1.25 (0.51–3.04)	0.622	1.80 (0.82–3.94)	0.143
>12 weeks	43	1		1	
Advice from a medical professional					
yes	189	0.95 (0.46–1.98)	0.896	1.53 (0.78–2.97)	0.214
no	69	1		1	
More attention on diet during pregnancy					
yes	201	0.65 (0.30–1.41)	0.272	0.72 (0.36–1.47)	0.371
no	57	1		1	
Body weight gain during pregnancy					
more than optimal	100	1.66 (0.77–3.61)	0.198	1.21 (0.60–2.46)	0.590
normal	79	0.72 (0.29–1.79)	0.475	1.54 (0.75–3.14)	0.239
less than optimal	79	1		1	

aOR: adjusted odds ratio; CI: confidence interval; O3: omega-3 fatty acids.

**Table 11:** Predictors of appropriately/inappropriately using FA by multinomial logistic regression.

Predictors	*n*	Inappropriate FA Users (vs. Non-Users)		Appropriate FA Users (vs. Non-Users)	
		aOR (95% CI)	*p* Value	aOR (95% CI)	*p* Value
Parity					
first	106	1.32 (0.48–3.63)	0.596	3.16 (1.05–9.46)	0.040
second or more	141	1		1	
Previous spontaneous abortion					
yes	60	1.40 (0.43–4.52)	0.575	1.67 (0.46–6.03)	0.432
no	187	1		1	
First appearance at prenatal care					
≤12 weeks	208	2.65 (0.97–7.25)	0.057	4.70 (1.48–14.99)	0.009
>12 weeks	39	1		1	
Advice from a medical professional					
yes	181	1.54 (0.60–3.94)	0.366	1.57 (0.57–4.37)	0.386
no	66	1		1	
More attention on diet during pregnancy					
yes	195	1.09 (0.39–3.07)	0.874	1.40 (0.45–4.36)	0.562
no	52	1		1	
Body weight gain during pregnancy					
more than optimal	95	1.05 (0.37–2.97)	0.935	0.74 (0.24–2.25)	0.589
normal	74	1.42 (0.44–4.58)	0.554	1.24 (0.36–4.27)	0.731
less than optimal	78	1		1	

aOR: adjusted odds ratio; CI: confidence interval; FA: folic acid.

**Table 12:** Predictors of appropriately/inappropriately using overall micronutrients by multinomial logistic regression.

Predictors	*n*	Inappropriate Combined Supplement Users (vs. Non-Users)		Appropriate Combined Supplement Users (vs. Non-Users)	
		aOR (95% CI)	*p* Value	aOR (95% CI)	*p* Value
Parity					
first	103	1.99 (0.83–4.79)	0.126	5.23 (1.92–14.23)	0.001
second or more	138	1		1	
Previous spontaneous abortion					
yes	58	0.61 (0.25–1.49)	0.278	1.63 (0.59–4.51)	0.346
no	183	1		1	
First appearance at prenatal care					
≤12 weeks	203	3.57 (1.45–8.84)	0.006	3.75 (1.33–10.58)	0.012
>12 weeks	38	1		1	
Advice from a medical professional					
yes	177	0.62 (0.27–1.43)	0.260	1.53 (0.57–4.11)	0.394
no	64	1		1	
More attention on diet during pregnancy					
yes	190	1.56 (0.66–3.72)	0.315	1.22 (0.47–3.16)	0.689
no	51	1		1	
Body weight gain during pregnancy					
more than optimal	92	1.79 (0.78–4.11)	0.168	1.18 (0.47–2.96)	0.727
normal	73	3.18 (1.20–8.47)	0.020	2.93 (1.03–8.31)	0.044
less than optimal	76	1		1	

aOR: adjusted odds ratio; CI: confidence interval.

**Table 13:** The association of micronutrient supplementation with different adherence levels in pregnancy.

Adherence Levels	GDM		Gestational Hypertension	
	OR (95% CI)	*p* Value	OR (95% CI)	*p* Value
PMM				
non-users	ref.		ref.	
inappropriate users	2.04 (0.31–13.30)	0.456	0.71 (0.14–3.61)	0.682
appropriate users	3.03 (0.41–22.21)	0.276	1.35 (0.24–7.42)	0.734
VD				
non-users	ref.		ref.	
inappropriate users	0.47 (0.05–4.99)	0.533	1.12 (0.13–9.42)	0.917
appropriate users	0.50 (0.04–6.86)	0.603	1.06 (0.09–12.35)	0.964
FA				
non-users	ref.		ref.	
inappropriate users	2.80 (0.62–12.66)	0.182	0.52 (0.07–3.70)	0.514
appropriate users	3.62 (0.61–21.38)	0.155	0.21 (0.02–1.96)	0.169
O3				
non-users	ref.		ref.	
inappropriate users	0.76 (0.21–2.78)	0.682	1.16 (0.31–4.29)	0.826
appropriate users	0.06 (0.003–0.97)	0.047	0.59 (0.11–3.24)	0.543
Combined supplement intake				
non-users	ref.		ref.	
inappropriate users	0.39 (0.05–3.19)	0.381	2.99 (0.23–38.30)	0.400
appropriate users	5.36 (0.12–249.78)	0.392	4.28 (0.10–188.20)	0.451

OR: odds ratio; CI: confidence interval; PMM: prenatal multiple micronutrient supplements; O3: omega-3 fatty acids; VD: vitamin D; FA: folic acid.

**Table 14:** The association of micronutrient supplementation with different adherence levels on delivery and birth.

Adherence Levels	Preterm Birth		High Birth Weight		Inadequate Uterine Contraction		Cephalopelvic Disproportion		Abnormal Rupture of Membranes		Cesarean Section	
	OR (95% CI)	*p*	OR (95% CI)	*p*	OR (95% CI)	*p*	OR (95% CI)	*p*	OR (95% CI)	*p*	OR (95% CI)	*p*
PMM												
non-users	ref.		ref.		ref.		ref.		ref.		ref.	
inappropriate users	0.64 (0.19–2.11)	0.463	3.34 (0.35–31.56)	0.292	1.14 (0.29–4.45)	0.855	3.06 (0.30–31.46)	0.348	0.88 (0.27–2.86)	0.834	1.49 (0.51–4.35)	0.470
appropriate users	1.37 (0.41–4.54)	0.610	4.06 (0.30–55.50)	0.293	2.52 (0.63–10.10)	0.192	2.52 (0.17–36.97)	0.500	1.14 (0.34–3.81)	0.829	2.04 (0.65–6.41)	0.223
VD												
non-users	ref.		ref.		ref.		ref.		ref.		ref.	
inappropriate users	1.18 (0.27–5.13)	0.824	0.45 (0.03–7.29)	0.562	0.42 (0.09–2.05)	0.281	1.71 (0.05–54.91)	0.763	0.47 (0.11–1.96)	0.297	1.11 (0.28–4.33)	0.886
appropriate users	2.47 (0.46–13.24)	0.291	0.19 (0.01–5.56)	0.337	0.40 (0.06–2.46)	0.322	0.60 (0.01–34.61)	0.807	0.77 (0.15–3.94)	0.754	1.01 (0.21–4.88)	0.990
FA												
non-users	ref.		ref.		ref.		ref.		ref.		ref.	
inappropriate users	1.61 (0.51–5.07)	0.414	5.22 (0.52–52.89)	0.162	0.80 (0.22–2.95)	0.733	4.02 (0.34–47.61)	0.270	0.87 (0.27–2.79)	0.820	0.31 (0.10–0.94)	0.038
appropriate users	1.56 (0.41–6.00)	0.519	6.44 (0.47–88.43)	0.163	1.32 (0.30–5.90)	0.716	4.88 (0.30–79.78)	0.266	1.92 (0.49–7.46)	0.349	0.25 (0.07–0.92)	0.036
O3												
non-users	ref.		ref.		ref.		ref.		ref.		ref.	
inappropriate users	1.25 (0.47–3.36)	0.652	0.63 (0.17–2.35)	0.489	4.24 (1.55–11.62)	0.005	0.31 (0.05–2.00)	0.219	1.77 (0.70–4.48)	0.227	0.55 (0.23–1.30)	0.175
appropriate users	1.30 (0.40–4.18)	0.666	0.30 (0.04–2.36)	0.253	2.90 (0.89–9.49)	0.078	0.49 (0.06–4.34)	0.528	1.32 (0.43–4.10)	0.631	0.42 (0.15–1.21)	0.107
Combined supplement intake												
non-users	ref.		ref.		ref.		ref.		ref.		ref.	
inappropriate users	0.57 (0.12–2.74)	0.484	0.63 (0.06–7.02)	0.708	1.25 (0.24–6.55)	0.794	0.19 (0.01–5.53)	0.335	1.58 (0.34–7.31)	0.560	1.46 (0.34–6.25)	0.610
appropriate users	0.25 (0.02–3.05)	0.279	1.08 (0.02–56.37)	0.970	0.67 (0.05–8.85)	0.759	0.99 (0.01–104.97)	0.996	0.73 (0.06–8.34)	0.797	2.59 (0.27–24.94)	0.411

OR: odds ratio, CI: confidence interval, PMM: prenatal multiple micronutrient supplements, O3: omega-3 fatty acids, VD: vitamin D, FA: folic acid.

## Data Availability

The dataset used and analyzed during the current study is available from the corresponding author on reasonable request due to the fact that other secondary analyses of the whole study database are still ongoing.

## Abbreviations

The following abbreviations are used in this manuscript:

FA	Folic acid
VD	Vitamin D
O3	Omega-3 fatty acids
PMM	Prenatal multiple micronutrient supplements
WHO	World Health Organization
GDM	Gestational diabetes mellitus
EPA	Eicosapentaenoic acid
DHA	Docosahexaenoic acid
OR	Odds ratio
CI	Confidence interval
aOR	Adjusted odds ratio
